# Real-World Data Assessing the Impact of Lymphovascular Space Invasion on the Diagnostic Performance of Sentinel Lymph Node Mapping in Endometrial Cancer

**DOI:** 10.3390/cancers16010067

**Published:** 2023-12-22

**Authors:** Carol A. Buechi, Franziska Siegenthaler, Laura Sahli, Andrea Papadia, Flurina A. M. Saner, Stefan Mohr, Tilman T. Rau, Wiebke Solass, Sara Imboden, Michael D. Mueller

**Affiliations:** 1 Department of Obstetrics and Gynecology, Bern University Hospital and University of Bern, 3010 Bern, Switzerland; carolanne.buechi@insel.ch (C.A.B.);; 2 Department of Gynecology and Obstetrics, Ente Ospedaliero Cantonale of Lugano, 6900 Lugano, Switzerland; 3 Facoltà di Scienze Biomediche, Università della Svizzera Italiana, 6900 Lugano, Switzerland; 4 Department of Gynecology and Obstetrics, Bürgerspital Solothurn, 4500 Solothurn, Switzerland; stefan.mohr@spital.so.ch; 5 Institute of Pathology, Universitätsklinikum Düsseldorf, 40225 Düsseldorf, Germany; 6 Institute of Tissue Medicine and Pathology, University of Bern, 3008 Bern, Switzerland

**Keywords:** endometrial cancer, sentinel lymph node mapping, lymphovascular space invasion

## Abstract

**Simple Summary:**

Sentinel lymph node (SLN) mapping has been introduced to endometrial cancer treatment as an alternative to lymph node dissection for lymph node staging. Lymphovascular space invasion (LVSI) is a known prognostic risk factor in endometrial cancer and is associated with lymph node metastasis and worse outcomes. This study shows that LVSI is an independent predictor of recurrence in node-negative endometrial cancer patients with SLN mapping alone. The negative predictive value of sentinel lymph node mapping is significantly lower in LVSI-positive endometrial cancer patients. Due to these findings, importance should be attached to LVSI in early-stage endometrial cancer patients with negative SLN mapping, and adjuvant therapy should be discussed more thoroughly in these cases.

**Abstract:**

Background: SLN mapping has emerged as a standard of care in endometrial cancer due to its high sensitivity and significant reduction in morbidity. Although lymphovascular space invasion (LVSI) is a known risk factor for lymph node metastasis and recurrence, evidence on the reliability of SLN mapping in LVSI-positive patients is scarce. The aim of this study was to determine the impact of LVSI on the diagnostic performance of SLN mapping. Methods: This retrospective cohort study included patients with endometrial cancer who underwent primary surgical treatment at the Bern University Hospital, Switzerland, between 2012 and 2022. Results: LVSI was present in 22% of patients and was significantly associated with lymph node metastasis (*p* < 0.001) and recurrence (*p* < 0.001). In node-negative patients with only SLN mapping performed, LVSI was an independent predictor of recurrence during multivariable Cox regression analysis (*p* = 0.036). The negative predictive value of SLN mapping was 91.5% and was significantly lower in tumors with LVSI (75.0%) compared to LVSI-negative tumors (95.6%, *p* = 0.004). Conclusion: The presence of LVSI was significantly associated with worse oncological outcomes. LVSI was an independent predictor of recurrence in node-negative patients with only SLN mapping performed. Furthermore, the negative predictive value of SLN mapping was significantly lower in LVSI-positive tumors.

## 1. Introduction

In developed countries, endometrial cancer is the most common gynecological cancer, with a 5-year survival rate of 80% [1]. The majority of endometrial cancer patients are diagnosed with early-stage disease, leading to a favorable prognosis. However, if there is regional involvement or even distant metastasis, the risk of recurrence and mortality remains high [2].

Lymph nodal status is one of the most important prognostic factors driving the adjuvant treatment of endometrial cancer. However, two randomized trials failed to show a survival benefit of the use of systematic lymphadenectomy in this disease [3,4], and complete lymph node staging has well-known morbidity, such as longer operation times, greater blood loss, and a higher rate of intra- and postoperative complications [5,6,7]. During the last decade, the treatment approach for endometrial cancer has become more personalized. One of the most important milestones in the de-escalation of surgical treatment is sentinel lymph node (SLN) mapping. Minimally invasive SLN mapping with indocyanine green (ICG) has excellent detection rates and high sensitivity [8,9,10]. ICG SLN mapping provides surgical nodal assessment, which is indispensable for determining the treatment strategy, together with a significantly decreased risk of morbidity compared to a complete lymphadenectomy [7,11]. ICG shows equivalent detection rates compared to other tracers such as blue dyes and technetium. Due to its safety profile and easy application, ICG has become the preferred detection technique for SLN mapping in endometrial cancer. According to the literature, SLN mapping has been introduced as an alternative to lymph node dissection for lymph node staging [12] and is now integrated as the standard of care in the 2021 European Society of Gynecological Oncology (ESGO), the European Society for Radiotherapy and Oncology (ESTRO), and the European Society of Pathology (ESP) guidelines for the management of patients with endometrial carcinoma [13]. If performed according to state-of-the-art principles, a negative sentinel node is accepted as confirmation of pN0 [13].

Several histopathological factors are taken into consideration to assess the risk of lymph node involvement in endometrial cancer. Of these, lymphovascular space invasion (LVSI) is well known as being associated with lymph node metastasis and worse oncological outcomes [14,15,16]. Furthermore, LVSI is even an independent predictor of survival, after adjustment for the presence of lymph node metastasis [17], and independently affects therapy recommendations for adjuvant treatment [13].

While the literature shows that LVSI is not associated with failed SLN mapping [12,18], evidence on the reliability of SLN mapping in patients with LVSI-positive endometrial cancer is scarce. The aim of this study was to determine the impact of LVSI on the diagnostic performance of SLN mapping.

## 2. Materials and Methods

### 2.1. Patient Cohort and Clinicopathological Data

This retrospective cohort study includes endometrial cancer patients who underwent primary surgical treatment, including ICG SLN mapping, between 2002 and 2012 at the Bern University Hospital. The experimental protocol was approved by the Ethics Commission of the Canton of Bern, Switzerland; it meets the guidelines of the responsible governmental agency. All patients signed written general consent forms for the use of their clinical data for research purposes. Demographic, clinical, and intraoperative data were retrieved from an electronic database. Follow-up data on recurrence and survival were available through standardized databases and follow-up controls. At the time of the final histopathological analysis, complete ultrastaging was performed in all sentinel lymph nodes by board-certified pathologists. Macrometastasis is defined as >2 mm the largest diameter focus of metastatic disease per lymph node, micrometastasis 0.2–2 mm, and isolated tumor cells ≤0.2 mm.

### 2.2. Surgical Procedure

At the beginning of surgery, one vial of 25 mg ICG powder (Pulsion^®^) was suspended in 10 mL of sterile water and an amount of 4–8 mL was injected into the cervix (submucosally and 1 cm deep in the stroma at the four cardinal points), in the vagina, or intratumorally. All study patients underwent minimally invasive staging surgery including near-infrared ICG SLN mapping followed by total hysterectomy and bilateral salpingo-oophorectomy. The indication for pelvic and para-aortic lymph node dissection was based on frozen section evaluation of the uterus and/or the SLN according to international guidelines [1,13,19]. All procedures were performed by gynecologic oncologists with extensive experience in minimally invasive surgery.

### 2.3. Outcomes

All patients received follow-up examinations according to international guidelines [1,13,19]. Recurrence-free survival was defined as the time from primary staging surgery to first recurrence or death due to any cause. Overall survival was defined as the time from primary staging surgery to death due to any cause. Patients who were alive were censored at the date of their last follow-up. Recurrences were classified into locoregional, abdominal, and distant, according to the first site of recurrence. For the calculation of the negative predictive value of SLN mapping, only patients with complete surgical lymph node staging were included.

### 2.4. Statistical Analysis

Statistical calculations were performed using the Statistical Package for Social Sciences (IBM SPSS Statistic Version 28.0.1.1). Categorical variables were reported as frequencies and percentages, and continuous variables were reported as means and standard deviations. Patient, tumor, and treatment characteristics were analyzed using Chi-squared statistics or Fisher’s exact test in the case of categorical variables and analysis of variance (ANOVA) for continuous variables. Survival curves were generated using the Kaplan–Meier method and compared using the log-rank test. Univariable Cox regression analyses were conducted to assess the relationship between the risk of recurrence and other prognostic factors. Any variables significant in the univariable analysis were included in the multivariable analysis. A *p*-value of less than 0.05 was considered statistically significant.

## 3. Results

### 3.1. General Characteristics of the Whole Study Cohort

During the study period, a total of 674 endometrial cancer patients underwent primary surgical treatment, of whom 466 underwent minimally invasive SLN mapping with ICG. The mean age at surgery was 65.7 years, and the mean BMI was 29.2 kg/m^2^. Approximately 87.8% of the study patients were postmenopausal. The majority of the patients underwent laparoscopic SLN mapping. Most of the patients had FIGO stage I disease (75.8%) and endometrioid histology (81.3%). LVSI was present in 21.7% of the patients and blood vessel invasion was present in 9.7%. Approximately 14.8% of all patients presented with lymph node metastasis. A total of 247 (53.0%) patients received adjuvant treatment consisting of vaginal brachytherapy (26.0%), chemoradiotherapy (23.0%), chemotherapy (3.0%), external beam radiotherapy (0.9%), and endocrine therapy (one patient). Table 1 presents a detailed description of the main clinicopathological characteristics of the study cohort.

### 3.2. Clinicopathological Characteristics of Tumors with Lymphovascular Space Invasion

LVSI was present in 101 of the study patients (21.7%), absent in 359 (77.0%), and not assessed in six (1.3%). LVSI was significantly associated with a higher age at diagnosis and lymph node metastasis. LVSI-positive tumors showed further unfavorable pathological characteristics such as non-endometrioid histology and deep myometrial invasion. A more detailed description of the clinicopathological characteristics of LVSI-positive and -negative patients is provided in Table 2.

### 3.3. Performance of ICG SLN Mapping

ICG was injected into the cervix in 91.0% of the study patients, into the vagina in 2.1%, and hysteroscopically intratumorally in 6.9%. The overall SLN detection rates were 97.4% in the whole study cohort, 97.2% in LVSI-negative tumors, and 98.0% in LVSI-positive tumors. Table 3 shows a more detailed description of the performance of ICG SLN mapping. In total, 1824 SLNs were removed, with a mean number of 3.9 per patient. Overall, 58 patients showed sentinel lymph node metastasis, consisting of macrometastasis in 41 patients (8.8%), micrometastasis in 14 patients (3.0%), and isolated tumor cells in 3 patients (0.6%). Isolated para-aortic lymph node metastasis was seen in 14 patients (3%) and presented more often in LVSI-positive patients (10.9%) compared to LVSI-negative patients (0.8%) (*p* < 0.001). A total of 186 patients had successful SLN mapping and additional pelvic and/or para-aortic lymph node staging performed. Of these, 12 patients had a false-negative SLN, of whom 7 showed isolated para-aortic lymph node metastasis. The negative predictive value was 91.5% overall but was significantly lower in patients with evidence of LVSI (75.0%) compared to LVSI-negative cases (95.6%, *p* = 0.004). More details are provided in the Supplementary Materials (Tables S1–S3).

### 3.4. Oncological Outcome

**Figure 1:**
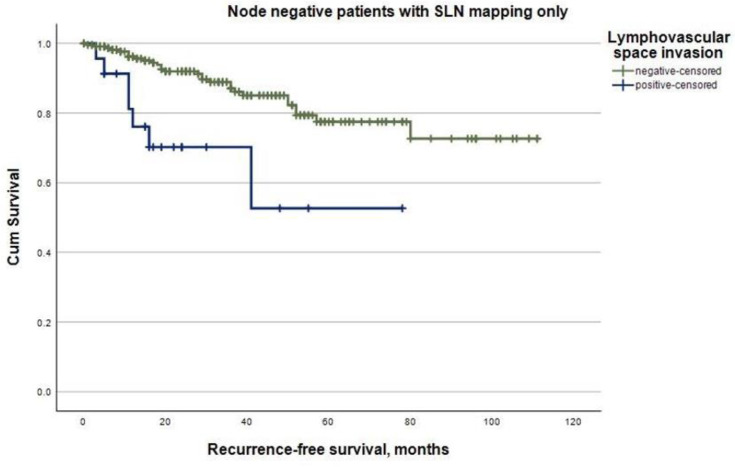
Recurrence-free survival according to lymphovascular space invasion in patients with node-negative endometrial cancer with sentinel lymph node mapping only performed (log-rank, *p* < 0.001).

**Figure 2:**
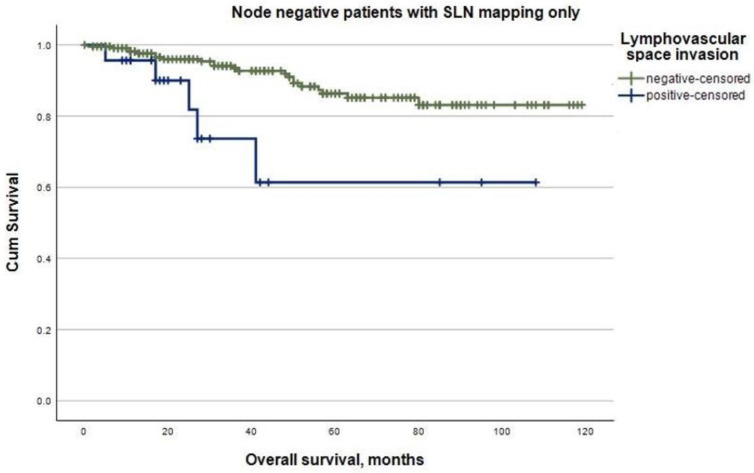
Overall survival according to lymphovascular space invasion in patients with node-negative endometrial cancer with sentinel lymph node mapping only performed (log-rank, *p* = 0.004).

The mean follow-up was 53.1 months (95% CI 50.0–56.3) for the whole study cohort. A total of 78 (16.7%) patients died, and 53 (11.4%) experienced at least one recurrence during follow-up. In total, there were 16 (30.2%) locoregional, 9 (17.0%) abdominal, and 28 (52.8%) distant recurrences. The recurrence rates were significantly higher in patients with LVSI-positive tumors (22.0%) compared to patients with LVSI-negative endometrial cancer (8.6%) (*p* < 0.001). Regarding node-negative endometrial cancer, in patients with only SLN mapping performed, evidence of LVSI was significantly associated with shorter mean recurrence-free survival (51.3 months, 95% CI 35.1–67.5) compared to patients with LVSI-negative tumors (91.8 months, 95% CI 85.3–98.4) (log-rank, *p* < 0.001, Figure 1). Similar associations were seen in mean overall survival, with it amounting to 76.8 months (95% CI 54.0–99.5) for patients with LVSI-positive tumors and 106.2 months (95% CI 101.2–111.2) for patients with LVSI-negative tumors (log-rank, *p* = 0.004, Figure 2). In this subgroup, LVSI was an independent predictor of recurrence (HR 3.5, 95% CI 1.1–11.3, *p* = 0.036) in the multivariable Cox regression analysis, including tumor stage and grading (Table 4). In contrast, in node-negative endometrial cancer patients with an additional pelvic and/or para-aortic lymph node dissection performed, LVSI was not associated with the risk of recurrence in the univariable Cox regression analysis (HR 1.7, 95% CI 0.6–5.1, *p* = 0.346). Furthermore, in patients with histologically confirmed lymph node metastases, LVSI was not a predictor of recurrence in the univariable Cox regression analysis (HR 2.2, 95% CI 0.5–9.9, *p* = 0.287).

## 4. Discussion

### 4.1. Summary of the Main Results

In this retrospective, single-center cohort study, we evaluated the impact of lymphovascular space invasion on the diagnostic performance of sentinel lymph node mapping in endometrial cancer. In our study cohort, SLN mapping showed unilateral and bilateral detection rates of 91.0% and 97.4%, respectively.

LVSI was present in 22% of the study patients and was significantly associated with lymph node metastasis and recurrence. The negative predictive value of SLN mapping was 91.5% overall and significantly lower in tumors with evidence of LVSI. Furthermore, in node-negative patients with only SLN mapping performed, LVSI was an independent predictor of recurrence in the multivariable Cox regression analysis, including tumor stage and grading.

### 4.2. Results in the Context of the Published Literature

Consistent with the current literature, our study results showed excellent bilateral and unilateral detection rates for SLN mapping with ICG in endometrial cancer [8,10,20]. As previously described, the failure of SLN mapping with ICG is associated with advanced tumor stage, enlarged lymph nodes, and lymph node involvement but not with LVSI [12,18]. This is also reflected in our population with similar SLN detection rates in LVSI-negative and LVSI-positive tumors. In line with the current literature, 22% of our study patients showed the presence of LVSI, which was associated with lymph node metastasis and unfavorable pathological characteristics such as non-endometrioid histology and deep myometrial invasion [17,21,22,23]. Of our study patients, 15% presented with lymph node metastasis, of whom 20% had isolated para-aortic lymph node involvement, accounting for 3% of the whole study cohort—completely in line with previous studies [24,25,26,27]. In our cohort, isolated para-aortic lymph node metastasis was present significantly more often in LVSI-positive tumors, as described before by Chang et al. [28]. Furthermore, according to our results, almost two-thirds of the patients with a false-negative SLN presented with isolated para-aortic lymph node metastasis. The negative predictive value of SLN mapping was 91.5% overall, which was below the previously reported values of the FIRES trial (99.6%) [7]. On the one hand, this difference could be explained by the different study populations (while the FIRES trial only included early-stage tumors, our cohort also consisted of the advanced stages); on the other hand, para-aortic lymph node dissection was performed in only 58% of the study patients of the FIRES trial, potentially missing some of the isolated para-aortic lymph node metastasis and therefore underestimating the false-negative rate. Furthermore, in our study, only patients with histopathological risk factors underwent complete lymph node dissection. Therefore, the assessment of the diagnostic performance of SLN mapping in this study was performed on a subgroup of mainly high-risk endometrial cancer patients. Our results showed a significant decrease in the negative predictive value of SLN mapping in LVSI-positive tumors. This association has not been investigated in the literature previously. In our opinion, a possible explanation for these findings is the more frequent presence of isolated para-aortic lymph node metastases in LVSI-positive tumors [28]. LVSI was associated with negative oncological outcomes in our endometrial cancer study patients, as previously described [29,30]. In our multivariable Cox regression analysis, this association remained evident only in the subgroup of node-negative patients with SLN mapping alone. In our opinion, this might be partly explained by the poorer diagnostic performance of SLN mapping in LVSI-positive tumors and the risk of missing lymph node metastases. On the other hand, LVSI is described as an independent predictor of survival after adjustment for the presence of lymph node metastasis [17,31].

### 4.3. Strengths and Weaknesses

To the best of our knowledge, this is the first study analyzing the impact of LVSI on the reliability of SLN mapping in endometrial cancer patients. The major strengths of our study include the long follow-up period and the large sample size. The most important limitations are the retrospective study design and the missing substratification of LVSI into “focal” and “substantial” groupings. Furthermore, complete lymph node staging was performed only in the subgroup of patients with unfavorable histopathological features. As our study period goes back to 2012, more than half of our study patients lack data on the molecular classification of the tumor, which is why the molecular subgroups were not evaluated further.

### 4.4. Implications for Practice and Future Research

One of the most important milestones in the de-escalation of surgical treatment is SLN mapping, which was implemented as a standard of care in the recent ESGO/ESTRO/ESP recommendations [13]. While SLN mapping showed excellent detection rates and high sensitivity [8,9,10,20], in previous studies, we could demonstrate a significantly lower negative predictive value in the case of the presence of LVSI: in one out of four LVSI-positive tumors, lymph node involvement might be missed due to a false-negative SLN. Nonetheless, these results must be interpreted with caution, since only a subgroup of patients with high-risk tumors underwent comprehensive surgical lymph node staging in this study and were therefore included in these calculations. On the other hand, the rather low-risk subgroup of node-negative patients who underwent SLN mapping alone had a significantly worse prognosis in the case of the presence of LVSI; we can only speculate as to whether or not this is due to a false-negative SLN. Isolated para-aortic lymph node metastasis might be a possible explanation for our results as they were more frequent in LVSI-positive tumors and contributed to a large proportion of the false-negative SLNs. In applying these results in clinical practice, we should turn our attention to the presacral SLNs as possible representatives of the lower paracervical pathway with further drainage to the paraaortic area [32]. On the other hand, adjuvant therapy should be discussed more thoroughly in early-stage endometrial cancer patients with LVSI and SLN mapping performed only. A recently published review has demonstrated that adjuvant treatment improves overall survival in women with high-intermediate risk early-stage endometrial cancer with LVSI [33]. In our opinion, it would be ill-advised to recommend complete lymph node dissection in all early-stage low-risk endometrial cancer patients with LVSI. That said, the staging and treatment of endometrial cancer should be discussed in a multi-disciplinary setting, and patients should be involved in and counseled about different treatment opportunities. LVSI is already treated as a prognostic risk factor, and it should be taken into consideration, particularly in cases with negative SLN mapping.

## 5. Conclusions

In our study of patients with endometrial cancer, the presence of LVSI was significantly associated with lymph node metastasis, advanced tumor stage, non-endometrioid histology, and high-grade tumors. The oncological outcome was significantly worse in patients with LVSI-positive tumors. In the subgroup of patients with node-negative endometrial cancer undergoing SLN mapping only, LVSI was an independent predictor of recurrence in the multivariable Cox regression analysis. Furthermore, the negative predictive value of SLN mapping was significantly lower in tumors with evidence of LVSI.

## Figures and Tables

**Table 1:** Patients’ demographics and surgical and histological baseline characteristics.

Whole Study Cohort (*n* = 466)	
Mean age, years ± SD	65.7 ± 11.3
Mean BMI, kg/m^2^ ± SD	29.2 ± 7.5
Menopausal status, *n* (%)	
- premenopausal	55 (11.8)
- postmenopausal	409 (87.8)
- unknown	2 (0.4)
Surgical approach, *n* (%)	
- laparoscopic	449 (96.4)
- robotic	2 (0.4)
- conversion to laparotomy	15 (3.2)
FIGO stage, *n* (%)	
- I	353 (75.8)
- II	21 (4.5)
- III	87 (18.7)
- IV	5 (1.1)
Grading, *n* (%)	
- G1	160 (34.3)
- G2	165 (35.4)
- G3	141 (30.3)
Histological subtype, *n* (%)	
- endometrioid	379 (81.3)
- serous	29 (6.2)
- clear cell	11 (2.4)
- undifferentiated	2 (0.4)
- carcinosarcoma	21 (4.5)
- mixed	23 (4.9)
- neuroendocrine	1 (0.2)
Lymphovascular space invasion, *n* (%)	
- present	101 (21.7)
- absent	359 (77.0)
- missing	6 (1.3)
Blood vessel invasion, *n* (%)	
- present	45 (9.7)
- absent	415 (89.1)
- missing	6 (1.3)
Lymph node metastasis, *n* (%)	
- present	69 (14.8)
- absent	392 (84.1)
- not assessed	5 (1.1)
Adjuvant treatment, *n* (%)	
- none	219 (47.0)
- external beam radiotherapy	4 (0.9)
- vaginal brachytherapy	121 (26.0)
- chemoradiotherapy	107 (23.0)
- chemotherapy	14 (3.0)
- endocrine therapy	1 (0.2)

Abbreviations: *n* = number, SD = standard deviation, BMI = body mass index, and FIGO = Federation International de Gynecologie et Obstetrique.

**Table 2:** Clinicopathological characteristics of endometrial cancer patients according to lymphovascular space invasion.

	LVSI Negative (*n* = 359)	LVSI Positive (*n* = 101)	*p*-Value ^a^
Mean age, years ± SD	64.9 ± 11.2	69.3 ± 10.6	**<0.001**
Advanced FIGO stage, *n* (%)	31 (8.6)	61 (60.4)	**<0.001**
High-grade tumors, *n* (%)	90 (25.1)	50 (49.5)	**<0.001**
Tumor size, mm ± SD	27.1 ± 18.6	47.9 ± 24.5	**<0.001**
Endometrioid histology, *n* (%)	301 (83.8)	71 (70.3)	**0.002**
Deep myometrial invasion (>50%), *n* (%)	98 (27.3)	72 (71.3)	**<0.001**
Lymph node metastasis, *n* (%)	13 (3.6)	56 (55.4)	**<0.001**
Adjuvant treatment, *n* (%)	157 (43.7)	90 (89.1)	**<0.001**

Abbreviations: *n* = number, SD = standard deviation, FIGO = Federation International de Gynecologie et Obstetrique, and LVSI = lymphovascular space invasion. ^a^ *p*-values reflect χ^2^ or Fisher’s exact test for categorical variables and ANOVA for continuous variables. A statistically significant *p*-value lower than 0.05 is marked in bold in the table.

**Table 3:** Performance of indocyanine green sentinel lymph node mapping in the whole study cohort and among patients with and without lymphovascular space invasion.

	Whole Study Cohort (*n* = 466)	LVSI Negative (*n* = 359)	LVSI Positive (*n* = 101)	*p*-Value
SLN mapping, *n* (%)				
- bilateral	424 (91.0)	325 (90.5)	93 (92.1)	
- unilateral	30 (6.4)	24 (6.7)	6 (5.9)	
- failed	12 (2.6)	10 (2.8)	2 (2.0)	0.868
Number of sentinel lymph nodes removed, mean ± SD	3.9 ± 2.5	3.9 ± 2.5	4.0 ± 2.5	0.604
LN staging performed, *n* (%)				
- SLN mapping alone	278 (59.7)	240 (66.9)	32 (31.7)	
- additional radical pelvic and/or para-aortic lymph node dissection	188 (40.3)	119 (33.1)	69 (68.3)	**<0.001**
SLN metastasis, *n* (%)				
- negative	396 (85.0)	340 (94.7)	50 (49.5)	
- isolated tumor cells	3 (0.6)	2 (0.6)	1 (1.0)	
- micrometastasis	14 (3.0)	2 (0.6)	12 (11.9)	
- macrometastasis	41 (8.8)	5 (1.4)	36 (35.6)	
- missing	12 (2.6)	10 (2.8)	2 (2.0)	**<0.001**
Isolated para-aortic lymph node metastasis, *n* (%)	14 (3.0)	3 (0.8)	11 (10.9)	**<0.001**

Abbreviations: *n* = number, SD = standard deviation, and LVSI = lymphovascular space invasion. *p*-values reflect χ^2^ or Fisher’s exact test for categorical variables and ANOVA for continuous variables. A statistically significant *p*-value lower than 0.05 is marked in bold in the table.

**Table 4:** Cox regression for multivariable analysis of the risk of recurrence according to lymphovascular space invasion in node-negative endometrial cancer patients with only SLN mappingperformed.

Clinicopathological Factor	*p*-Value	HR	95% CI
Lymphovascular space invasion	**0.036**		
No (ref)		1.0	Reference
Yes		3.480	1.073–11.289
Grading	0.415		
Low grade (G1, G2) (ref)		1.0	Reference
High grade (G3)		2.516	0.273–23.191
Tumor stage	**0.026**		
Early stage (FIGO I and II) (ref)		1.0	Reference
Advanced stage (FIGO III and IV)		6.926	1.262–38.002

Abbreviations: HR = hazard ratio, CI = confidence interval, and FIGO = FIGO Federation International de Gynecologie et Obstetrique. A statistically significant *p*-value lower than 0.05 is marked in bold in the table.

## Data Availability

The data presented in this study are available from the corresponding author upon request.

## Supplementary Materials

The following supporting information can be downloaded at: https://www.mdpi.com/article/10.3390/cancers16010067/s1, Table S1: Calculation of positive predictive value, sensitivity, specificity, and negative predictive value for sentinel lymph node mapping including all patients with sentinel lymph node mapping and additional pelvic and/or paraaortic lymph node dissection performed; Table S2: Calculation of positive predictive value, sensitivity, specificity, and negative predictive value for sentinel lymph node mapping including all patients with lymphovascular space invasion, with sentinel lymph node mapping and additional pelvic and/or paraaortic lymph node dissection performed; Table S3: Calculation of positive predictive value, sensitivity, specificity, and negative predictive value for sentinel lymph node mapping including all patients without lymphovascular space invasion, with sentinel lymph node mapping and additional pelvic and/or paraaortic lymph node dissection performed.
