# Supplementary material for: Real-World Data Assessing the Impact of Lymphovascular Space Invasion on the Diagnostic Performance of Sentinel Lymph Node Mapping in Endometrial Cancer
Source: Cancers (Basel). 2023 Dec 22;16(1):67. doi: 10.3390/cancers16010067 (PMC10778553; doi:10.3390/cancers16010067)
Supplement: Supplementary file 1 [file cancers-16-00067-s001.zip › cancers-2782360-supplementary.pdf]

Supplementary materials

Table S1: Calculation of positive predictive value, sensitivity, specificity, negative predictive value for sentinel lymph node mapping including all patients with sentinel lymph node mapping and additional pelvic and/or paraaortic lymph node dissection performed.

n= 186	N1 (n= 56)	N0 (n= 130)	Predictive Value
SLN positive (n= 44)	44	0	PPV = 100%
SLN negative (n= 142)	12	130	NPV = 91.5%
Sensitivity & Specificity	Sensitivity = 78.6%	Specificity = 100%	

Abbreviations: n= Number; SLN= sentinel lymph node; PPV= positive predictive value; NPV= negative predictive value

Table S2: Calculation of positive predictive value, sensitivity, specificity, negative predictive value for sentinel lymph node mapping including all patients with lymphovascular space invasion, with sentinel lymph node mapping and additional pelvic and/or paraaortic lymph node dissection performed.

n= 68	N1 (n= 47)	N0 (n= 21)	Predictive Value
SLN positive (n= 40)	40	0	PPV =100%
SLN negative (n= 28)	7	21	NPV = 75.0%
Sensitivity & Specificity	Sensitivity = 85.1%	Specificity = 100%	

Abbreviations: n= Number; SLN= sentinel lymph node; PPV= positive predictive value; NPV= negative predictive value

Table S3: Calculation of positive predictive value, sensitivity, specificity, negative predictive value for sentinel lymph node mapping including all patients without lymphovascular space invasion, with sentinel lymph node mapping and additional pelvic and/or paraaortic lymph node dissection performed.

n= 118	N1 (n= 9)	N0 (n= 109)	Predictive Value
SLN positive (n= 4)	4	0	PPV =100%
SLN negative (n= 114)	5	109	NPV = 95.6%
Sensitivity & Specificity	Sensitivity = 44.4%	Specificity = 100%	

Abbreviations: n= Number; SLN= sentinel lymph node; PPV= positive predictive value; NPV= negative predictive value
